# Dielectric, Thermal and Water Absorption Properties of Some EPDM/Flax Fiber Composites

**DOI:** 10.3390/polym13152555

**Published:** 2021-07-31

**Authors:** Anton Airinei, Mihai Asandulesa, Maria Daniela Stelescu, Niţǎ Tudorachi, Nicusor Fifere, Adrian Bele, Valentina Musteata

**Affiliations:** 1 Petru Poni Institute of Macromolecular Chemistry, 41A Grigore Ghica Voda Alley, 700487 Iasi, Romania; ntudor@icmpp.ro (N.T.); fifere.nicusor@icmpp.ro (N.F.); bele.adrian@icmpp.ro (A.B.); valentina.musteata@icmpp.ro (V.M.); 2 National Research and Development Institute for Textile and Leather, Leather and Footwear Institute, 93 Ion Minulescu Street, 031215 Bucharest, Romania; maria.stelescu@icpi.ro

**Keywords:** EPDM rubber, dielectric properties, water uptake, relaxation, thermal stability, IR/TG/MS

## Abstract

This paper deals with the dielectric and sorption properties of some flax fiber-reinforced ethylene-propylene-diene monomer (EPDM) composites containing different fiber loadings as well as their behavior after exposure to different doses of electron beam irradiation. Three relaxation processes were evinced, a weak relaxation β at sub-T_g_ temperatures and two α-type relaxations above the T_g_. The EPDM/flax composites exhibited higher values of dielectric constant, dielectric loss and conductivity as compared to a pristine EPDM sample. Using thermogravimetric analysis (TG) coupled with Fourier transform infrared spectroscopy (FTIR) and mass spectrometry (MS) (TG/FTIR/MS system), the degradation products can be identified. The water uptake increased as the flax fiber level increased in composites. The water uptake tests of irradiated composites showed that the highest water content was obtained for a flax fiber level of 20 phr.

## 1. Introduction

Ethylene propylene diene terpolymer (EPDM) is a widely investigated commercially available elastomer. EPDM consists of ethylene and propylene units which provide a chemically saturated and stable backbone and the third monomer is a unconjugated diene. The polymerization reaction can be performed in a controlled way in order to keep the saturated backbone and to place the reactive unsaturation in a side chain where it is available for chemical modifications or vulcanization [1,2]. Due to the presence of the propylene unit, EPDM exhibits high resistance to heat, ozone, weather and high energy radiation. EPDM rubber also possesses flexibility at low temperatures due to the presence of the double bonds, good chemical stability, mainly to polar media, and excellent electrical insulation properties [2,3,4,5,6].

EPDM rubber has several uses in the automotive industry (profiles, hoses, seals), electrical and construction industries, aerospace or nuclear technology. EPDM is also utilized as a dielectric material for cable and wire insulation, especially cables in nuclear power plants which are subjected to thermal and radiation attacks during their utilization, and as an insulator matrix in rocket combustion chambers due to its excellent mechanical, chemical and electrical characteristics [7,8,9,10].

EPDM rubber can be vulcanized by peroxides or using a sulfur-accelerated system, which it is the most often used method for this material [3,4]. In comparison with the abovementioned conventional procedures used for vulcanization, radiation crosslinking under accelerated electron beams or γ-irradiation, affords some advantages because this process takes place at room temperature with different degrees of crosslinking, being a clean and fast process, which requires less energy and allows greater processing throughput [5,11,12,13]. Furthermore, the use of radiation crosslinking results in an improvement of the mechanical and electrical characteristics of the EPDM based-materials developed for specific requirements. The incorporation of various additives in the EPDM insulating formulations including reinforcing inorganic fillers, fibrous reinforcement, flame retardants, plasticizers also lead to an improvement of the electrical and mechanical properties of the resulting EPDM composites [2,3,14]. The enhancement in the properties of the reinforced composites can be determined by the higher interfacial interaction between filler and polymer matrix. Adding different levels of reinforcement materials matrix such as carbon fiber, aramid fiber, (nano)silica, PET fibers, carbon black, clay [6,15,16,17,18,19,20] to the EPDM can assure appropriate electrical properties of the composites and these materials can be profitably for applications in wires and cables subjected to various voltages.

Natural fibers can represent an alternative reinforcement for EPDM composites due to their advantages over carbon and glass fibers such as low production costs, less health risks, biodegradability, etc. [21,22]. From a literature survey, it can be found that few studies on the electrical properties of EPDM/natural fiber composites are reported, as well as the effect of irradiation on their properties. Thus, carrot foliage and corn gluten were used as reinforcing agents for EPDM and the dielectric and mechanical properties of these composites were discussed [23]. An enhancement of the mechanical and dielectric properties of some EPDM/rice husk composites designed as electrical insulators was reported [22]. The dielectric properties of some polypropylene and unsaturated polyester hybrid composites reinforced with bamboo/jute natural fibers were also analyzed taking into account various fiber loadings and ratios. According to the results, the type of polymer used appears to have little influence on the dielectric characteristics of the resulting composites [24].

The present work focused on the dielectric properties of some EPDM/flax composites in terms of dielectric constant, dielectric loss and conductivity. The effect of electron beam irradiation on the dielectric characteristics was analyzed with the respect to different flax loadings. The dielectric relaxation of the EPDM/flax composites was also discussed. The thermal stability of EPDM composites determined using the TG/FTIR/MS techniques and the water absorption properties were reported.

## 2. Materials and Methods

### 2.1. Materials

Ethylene propylene diene monomer (Nordel 4760), with Mooney viscosity of 70 ML_1+4_ at 120 °C, with an ethylene content of 70% and a density of 0.88 g/cm^3^ was supplied by Dow Chemical (Midland, MI, USA). The flax fibers were cut to a length of 3 mm. The features of the working materials presented in Table 1 were detailed in previous publications [4,14]. The EPDM/flax fiber composites were obtained by melt mixing on an electrically heated laboratory roll mill machine with a cooling system as described previously [4,14] using the following loadings of flax fibers: 0, 5, 10, 15 and 20 phr. The samples were labeled as E0, EF5, EF10, EF15 and EF20, respectively (Table 1).

### 2.2. Methods

Thermogravimetric analysis of EPDM composites was conducted in dynamic conditions on a STA 449F1 Jupiter instrument (Netzsch, Selb, Germany) in nitrogen atmosphere, at a heating rate of 10°/min in the temperature range of 30–700 °C. Samples of 10–15 mg were placed in Al_2_O_3_ crucibles, Al_2_O_3_ being the reference material. The released gases from the TGA instrument were analyzed using an online connected FTIR spectrometer (Vertex 70, Bruker, Ettlingen, Germany) equipped with an external module TGA-IR and an Aeolos QMS 403C mass spectrometer (Netzsch). The IR absorption spectra were obtained over the wavenumber range of 600–4000 cm^−1^ at a resolution of 4 cm^−1^. The FTIR transfer line of the evolved gases was made from polytetrafluoroethylene heated at 190 °C. The acquisition of FTIR spectra in 3D format was performed with the OPUS 6.5 software. The gas transfer line to mass spectrometer was made from a quartz capillary heated at 240 °C. QMS 403C spectrometer works at 10^−5^ mbar vacuum and electron impact ionization energy of 70 eV. The data acquisition was achieved in the range m/z of 1-300, measurement time of 0.5 s.

All sorption measurements were realized with an IGAsorp dynamic sorption analyzer (Hiden Analytical, Warrington, UK) equipped with an ultrasensitive microbalance taking into account the modifications in the sample mass against the temperature and relative humidity. Before sorption experiment, each sample was dried in nitrogen flow (250 mL/min) until the weight was in equilibrium at relative humidity (RH) < 1%. The measurements were made at 25 °C in a RH range of 0–90%, using humidity steps of 10%, each step having a pre-established equilibrium time between 5 and 10 min.

The measurement of dielectric properties of the EPDM/flax composites was carried out with a broadband dielectric spectrometer (BDS40 system, Novocontrol GmbH, Hundsangen, Germany). The spectrometer was equipped with an Alpha–A high frequency analyzer. The determinations were done over a frequency window of 1–10^6^ Hz in the temperature range from −120 °C to 120 °C. The measured geometry was a plate-capacitor formed by a disc shaped sample having 1 mm thickness and placed between two round gold-plated flat electrodes of 20 mm diameter of the test capacitor. All the samples were gold-coated on their both surfaces, in order to assure a good electrical contact between the sample surfaces and the electrodes used to carry the dielectric measurements.

Irradiation of the EPDM/flax composites was achieved on an ALIN10 electron beam accelerator at room temperature as described in [4]. Specimens of rectangular shape (100 mm × 100 mm × 2 mm) were exposed to electron beam irradiation at dosages of 75, 150, 300 and 600 kGy, respectively.

## 3. Results and Discussion

### 3.1. Dielectric Behavior

In the present study, two main aspects were followed by the dielectric technique: the modification of molecular mobility and the variation of the electrical resistivity for nonirradiated and electron beam irradiated EPDM composites as compared to the initial EPDM sample. The mobility in composites can be firstly affected by the physical immobilization of polymer chains imposed by the flax fibers and then, by the crosslinking process derived from the electron beam irradiation. It is known, that in the EPDM/carbon black composites, NMR relaxation experiments proved unambiguously the immobilization of EPDM chain fragments on the surface of carbon black filler [25]. In this case, physical junctions forming a semi-permanent network in the vicinity of carbon aggregates were evinced. The electrical properties of the composites consisting of two or more phases can also be different depending on the properties of phases. In addition, the presence of fillers in the host polymers creates the conditions for Maxwell-Wagner-Sillars (MWS) polarization due to the difference between the intrinsic electrical properties of the media [26,27,28].

#### 3.1.1. Evolution of Dielectric Parameters with Frequency and Temperature

**Figure 1:**
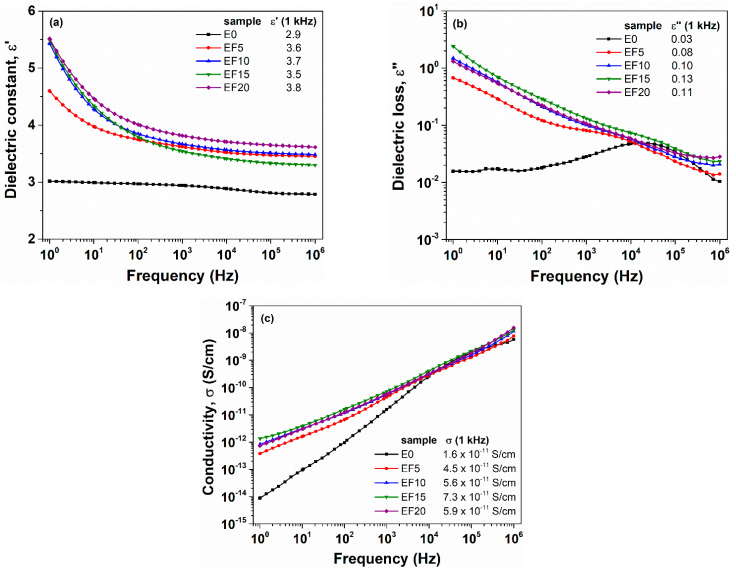
Comparative evolution of the dielectric constant (**a**), dielectric loss (**b**) and conductivity (**c**) as a function of applied field frequency for EPDM composites. The numerical values of dielectric parameters are retrieved in the inset of figures, at a frequency of 1 kHz.

The frequency dependences of the dielectric constant, ε′, dielectric loss, ε″ and conductivity, σ, at room temperature for EPDM sample without flax (E0) and EPDM/flax composites are given in Figure 1. It can be seen that ε′ decreases as the frequency increases and at higher frequencies it reaches a constant plateau (Figure 1a). At these frequencies the dipoles cannot orient sufficiently rapidly with the oscillations of the electrical field. Also, the orientation polarization decreases because of the restricted mobility of the charges. The numerical values of dielectric constant (Figure 1a inset) were derived from the plateau region, at a frequency of 1 kHz. The E0 sample exhibits the lowest magnitude of ε′ due to the low polarity of the neat polymer backbone [29]. Besides, the low value of dielectric constant for neat EPDM was observed by other authors [28,29]. The values of dielectric constant for EPDM/flax composites increase gradually with the flax loading. A similar conclusion was reported by Marin-Genesca and coworkers for ethylene propylene diene monomer blended with grinding recycled tire microparticles [28]. We believe that the reactive polar groups of the flax fibers cause an intense dipolar activity and consequently boost the dielectric constant of EPDM/flax composites. 

As follows from Figure 1b,c, the values of ε″ and σ are relatively low (at f = 1 kHz, below 0.1 and 10^−11^ S/cm, respectively), revealing the insulating character of these composites. In the low frequency range, the dielectric constant and dielectric loss have the highest values taking into account the free charges in composites [30]. This effect may be attributed to the interfacial polarization between the EPDM matrix and the flax fibers [31,32]. It is clear that the losses and the conductivity of composites increase slightly with an increasing amount of flax fibers.

**Figure 2:**
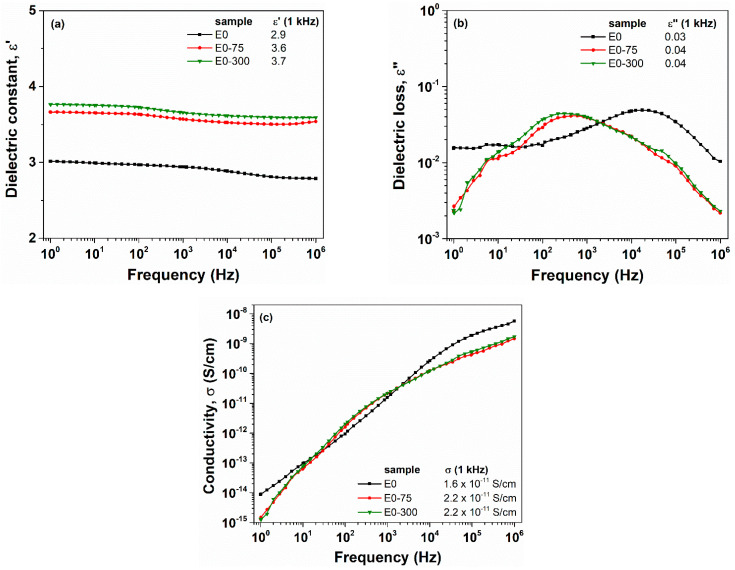
Evolution of the dielectric constant (**a**), dielectric loss (**b**) and conductivity (**c**) as a function of applied field frequency for E0 sample at different irradiation doses. The numerical values of dielectric parameters are retrieved in the inset of figures, at a frequency of 1 kHz.

**Figure 3:**
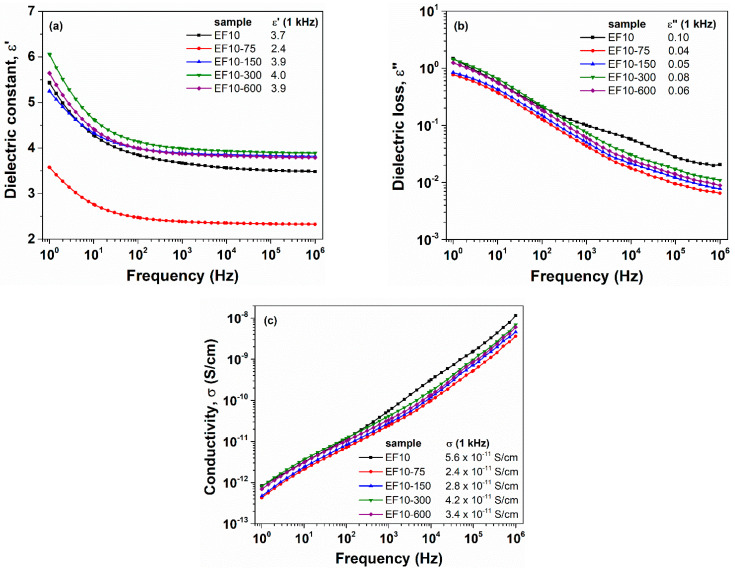
Evolution of the dielectric constant (**a**), dielectric loss (**b**) and conductivity (**c**) as a function of applied field frequency for EF10 sample at different irradiation doses. The numerical values of dielectric parameters are retrieved in the inset of figures, at a frequency of 1 kHz.

Figure 2 and Figure 3 show the frequency dependencies of ε′(a), ε″ (b) and σ (c) for E0 and EF10 samples, respectively, at different e-beam irradiation doses. Overall, we may notice that the dielectric constant of irradiated samples is enhanced as compared to non-irradiated sample (Figure 2a and Figure 3a). The effect of e-beam irradiation is remarkable for the pristine EPDM sample (E0), since ε′ increases from 2.9 (non-irradiated E0) up to 3.6 (E0-75 irradiated sample), providing, thus, an improvement of about 25%. However, for EF10 composite, only a slight improvement of dielectric constant was observed (about 6–8%). On the other hand, considering the Figure 2b,c, the effect of e-beam irradiation on ε″ and σ parameters of E0 sample is not clear. However, the dielectric loss and conductivity decrease for irradiated EF10 samples. In our previous work [4], we reported that the electron beam irradiation increased the crosslink density and reduced the crystallization degree of the samples. Following this premise, we may estimate that the improved crosslinking degree of the irradiated samples restricts the transport of charge carriers through the sample’s backbone and, consequently, the conductivity of the irradiated samples decreases.

**Figure 4:**
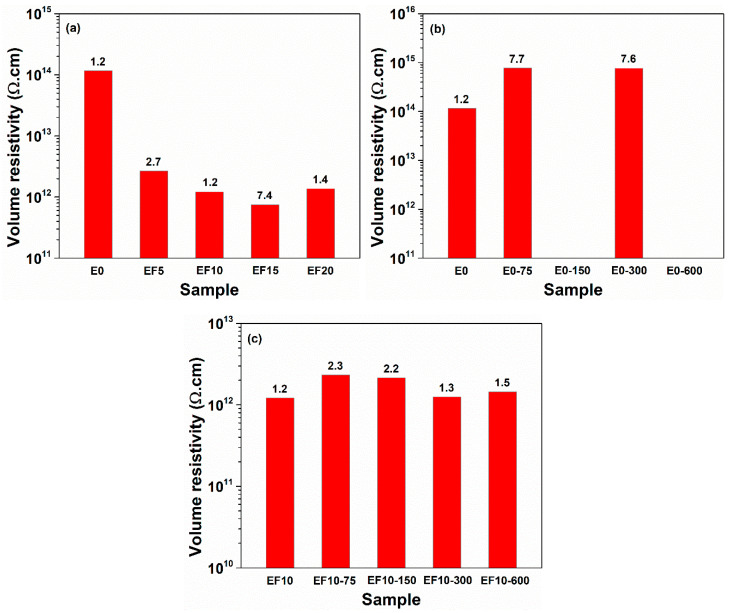
Volume resistivity of EPDM composites versus flax loading (**a**), and irradiation dose: E0 (**b**), and EF10 (**c**). The numerical values of volume resistivity for all considered samples are retrieved at the top of the bar chart, and at a frequency of 1 Hz.

Figure 4 illustrates a comparison between the values of volume resistivity measured at 1 Hz for E0 sample and EPDM/flax composites. The incorporation of the flax fibers in the composites determines a decrease of the resistivity even with two orders of magnitude for composites (Figure 4a). The electron beam irradiation applied to pristine EPDM causes the increase of the volume resistivity for irradiated samples (Figure 4b). On the other hand, the irradiated composite EF10 reveals a slight improvement of the insulating properties as a result of irradiation (Figure 4c). High volume resistivity values were also obtained for other EPDM-based composites [30].

**Figure 5:**
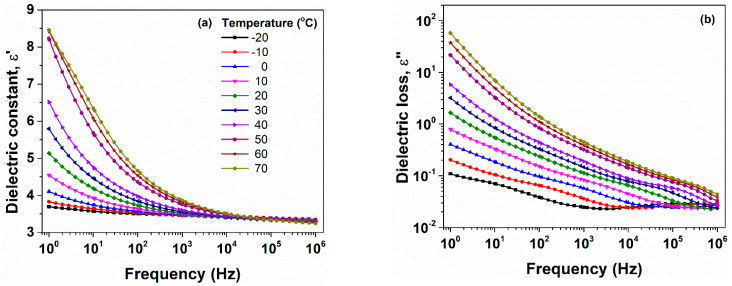
Exemplary evolution of dielectric constant (**a**) and dielectric loss (**b**) with frequency at various temperatures, for EF15 sample, as representative example. The legend provided in (**a**) should be considered for (**b**) as well.

In Figure 5, the isothermal plots of ε′ and ε″ as a function of frequency are presented for EF15 composite at selected temperatures. The other examined samples revealed a similar behavior. According to this figure, the magnitude of the dielectric parameters increases gradually together with the increase of the temperature, revealing some characteristic dipolar relaxation processes. The dielectric relaxations will be further presented.

**Figure 6:**
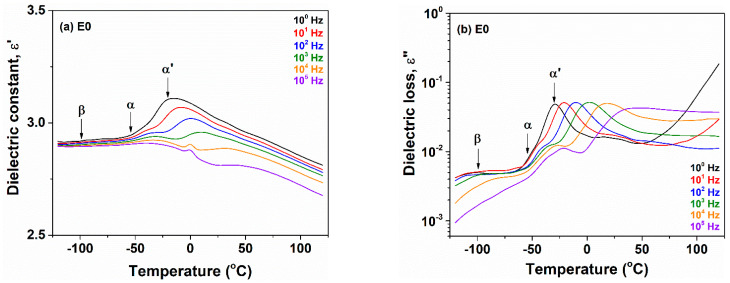
Evolution of dielectric constant (**a**) and dielectric loss (**b**) as a function of temperature at different frequencies for E0 sample. The arrows indicate the dielectric peaks of dipolar relaxations.

Figure 6 represents the dielectric behavior of the E0 sample in a temperature domain at fixed frequencies. The dielectric constant and dielectric loss reveal three dipolar relaxations as the temperature increases, noted as β, α and α′, respectively. They are observed like small step increase of ε′ and dielectric peaks or shoulders in ε″, shifting to higher temperatures with increasing frequency. The secondary β-relaxation peak can be associated with local motions appearing at lower temperatures than the primary-type α-relaxation [13]. Considering the EPDM structure and polarity, two scenarios for the secondary relaxations centered at −110 °C and up to −60 °C with increasing frequency can be taken into account. One possibility is given by the local movement of the diene side units. The second implies that this relaxation occurs by “crankshaft” type motion of the CH_2_ groups which may have attached some more polar impurities, similar to the low temperature γ-relaxation observed around −100 °C in polyurethanes [20,33].

**Figure 7:**
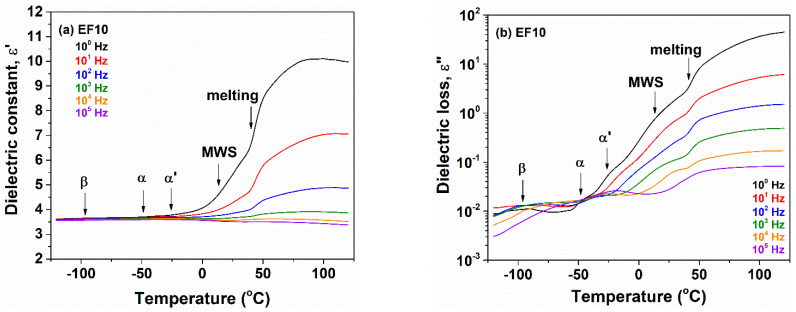
Plots of dielectric constant (**a**) and dielectric loss (**b**) as a function of temperature at different frequencies for nonirradiated EF10 composite.

**Figure 8:**
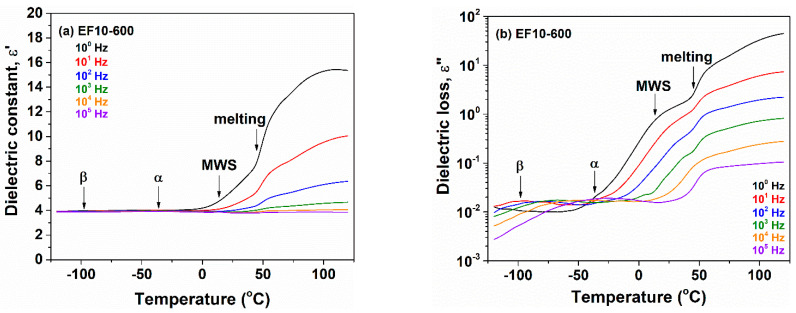
Plots of dielectric constant (**a**) and dielectric loss (**b**) as a function of temperature at different frequencies for irradiated EF10-600 composite.

The variation of dielectric constant and dielectric loss against temperature with respect to frequency is shown in Figure 7 for nonirradiated EF10 composite and in Figure 8 for irradiated at 600 kGy EPDM/flax composite EF10. For the composite EF10, the β-relaxation remains practically unchanged (Figure 7a,b), whereas for the electron beam irradiated composites this relaxation is widened and slightly shifted to higher temperatures (Figure 8a,b). This means that the relaxation is slower and it has a wider distribution of the relaxation times because the crosslinks restrict the mobility associated with this process.

With further increasing temperature, two dielectric relaxations were observed. They are correlated with the glass transition of EPDM chains located in the amorphous phase and among the crystallites, being designed as α and α′, respectively. For EF10 composite and the irradiated EF10-600 sample, the α and α′ dipolar relaxations are strongly overlapped in both the ε′ and ε″ representations. In addition, for composite-type materials, a frequency independent process due to the melting of crystallites is visible with further increases in temperature. This physical process is detected between 40 and 45 °C for the nonirradiated EF10 composite and 45–50 °C for the irradiated composite EF10-600. The melting process was previously observed by DSC thermograms between 34 and 46 °C for EF10 and 40–56 °C for irradiated samples, respectively, [4,14] confirming the dielectric data.

**Figure 9:**
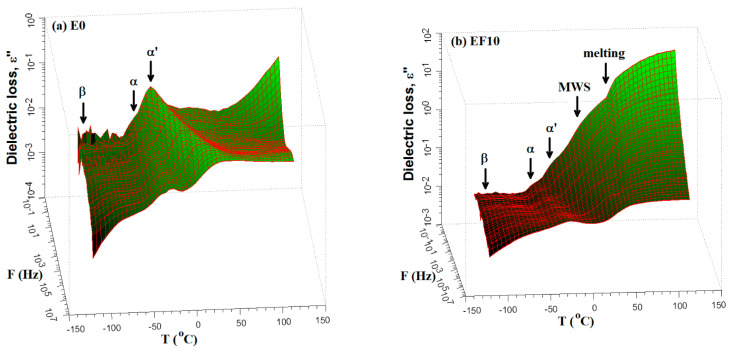

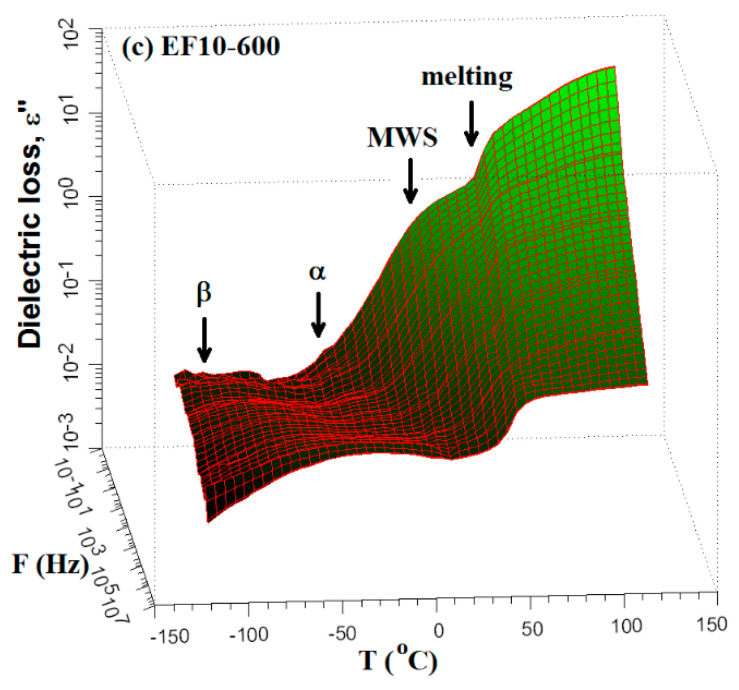
Variation of the dielectric loss with frequency and temperature for E0 (**a**), nonirradiated EF10 (**b**) and irradiated EF10-600 composites (**c**).

The 3D representations of the dielectric loss as a function of frequency and temperature are rpresented in Figure 9 for simple E0 sample, nonirradiated EF10 and irradiated EF10-600 composites. The diagrams allow the identification of dielectric α-, α′- and MWS-relaxations as well as the physical melting transition of crystallites. The broad band of the secondary β-relaxation is not clearly identified due to high noise presented at low temperatures.

#### 3.1.2. Segmental Relaxation

In this section, the detailed analyses of the segmental relaxation will be performed, following the interaction between polymer chains and flax fibers. For this reason, the ε″ (f) dependencies were processed based on the Havriliak-Negami (HN) relation:

(1) $$\varepsilon^{*}=\varepsilon \prime -i\varepsilon \prime \prime =\varepsilon_{\infty }+\frac{\Delta \varepsilon }{{\left[1+{\left({i\omega \tau }_{\mathrm{HN}}\right)}^{\alpha_{\mathrm{HN}}}\right]}^{\beta_{\mathrm{HN}}}}$$

where Δε is the relaxation strength of the dipolar process, ω = 2πf represents the angular frequency, f is the alternating field frequency, τ_HN_ is the HN relaxation time of the dipolar process, α_HN_ and β_HN_ are the shape-related parameters of the dielectric peak [34]. Furthermore, the specific relaxation time attributed to the peak maxima, τ_max_, was evaluated with the following expression:

(2) $$\tau_{\max}=\tau_{\mathrm{HN}}{\left[\frac{\sin\frac{\pi \mathrm{ab}}{2+2b}}{\sin\frac{\pi a}{2+2b}}\right]}^{\frac{1}{a}}$$

The main relaxation peaks, α and α′, are related to the cooperative reorientation motions of the main segments. As previously noticed, the α-relaxation exhibits as a dielectric peak with reduced intensity. It is strongly overlapped by the α′-relaxation and, consequently, its curvature cannot be exactly identified and processed. On the other hand, α′-relaxation appears as a well-shaped dielectric peak with high intensity. This signal will be further processed for simple E0 polymer, EF5, EF10 and EF15 nonirradiated composites and also for E0-75 and E0-300 irradiated composite samples. For other samples, we believe that the dielectric peak specific to α′-relaxation is suppressed by the crosslinking process. The activation energy of the segmental α′-relaxation, B, was finally determined with the Vogel-Fulcher-Tammann (VFT) expression:

(3) $$\tau_{\max}\left(T\right)=\tau_{0}\exp\left(\frac{B}{k(T-T_{V}}\right)$$

where, τ_0_ is a prefactor, k is the Boltzmann’s constant, T is the absolute temperature and T_v_ is the so-called Vogel temperature.

**Figure 10:**
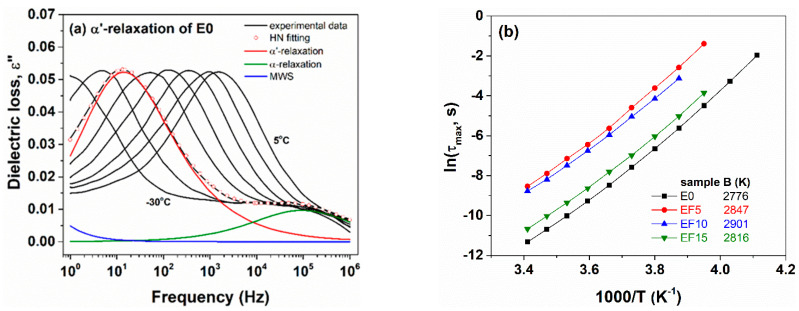
Representative separation of α′-relaxation for E0 sample (**a**) and Arrhenius plots for nonirradiated composites (**b**). In (**b**), lines are used to guide the eyes.

Figure 10a shows the isothermal plots of dielectric loss as a function of frequency for an E0 sample at selected temperatures between −30 °C and 30 °C. The well-shaped relaxation peak is shifted to higher frequencies as temperature increases, suggesting that the α′ dipolar transition is a thermally activated process. Following the Figure 10a, it is revealed that each ε″(f) dependency was fitted with two HN terms: the first one retrieved at low frequencies (maximum around 15 Hz) describes the dielectric peak of α′ relaxation and the second one observed at higher frequencies (maximum around 10^5^ Hz) related to the peak of α-relaxation. A supplementary term for the MWS process is limited at low frequencies. The dielectric peak of α′ relaxation was found asymmetric (β_HN_ ~0.7–0.8) and narrow (α_HN_ ~0.55–0.65). Closed values of β_HN_ and α_HN_ were also found for EF5, EF10 and EF15 nonirradiated composites.

The activation energy of E0 polymers and the corresponding composites are quite close (Figure 10b inset), suggesting that the content of flax fibers does not affect excessively the segmental molecular dynamics of the polymer. However, B values of nonirradiated composites are slightly lower than that of E0 polymer meaning that the flax fibers may reduce the rotational potential energy barrier for segmental motions of the polymer backbone. The results are in agreement with the thermal investigations of composites based on ethylene propylene diene rubber reinforced with flax fibers. In a previous work [14] we noticed that the T_g_ values of E0 and composites are comparable.

**Figure 11:**
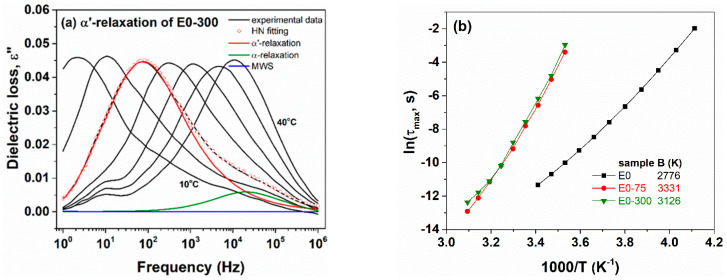
Representative separation of α′-relaxation for E0-300 sample (**a**) and Arrhenius plots for irradiated E0, E0-75 and E0-300 composites (**b**). In (**b**), lines are used to guide the eyes.

The ε″(f) dependencies of E0-300 sample at temperatures between 10 and 40 °C are displayed in Figure 11a. We notice that α′-relaxation of irradiated composites is also a thermally activated process, following the VFT equation (3). The deconvolution procedure is depicted in Figure 11a for the ε″(f) dependency presented at 20 °C. Here, the HN functions of α′ (depicted at 80 Hz), α (depicted around 3 × 10^4^ Hz) and a small term for the dielectric signal of MWS are enclosed. The HN shape parameters for α′-relaxation of E0-300 sample were obtained as β_HN_ ~0.75 and α_HN_ ~0.65 (on average over temperature), being similar with that of nonirradiated composites. However, according to Figure 11b, the values of activation energy of irradiated samples are higher than B value of sample E0 and, thus, of nonirradiated composites. It is clear that the crosslinking of composites due to e-beam irradiation restricted the segmental motions of the polymer backbone. The effect is in agreement with the previous findings by DSC, where the values of T_g_ for e-beam irradiated composites increased with the irradiation dose due to the reduced molecular mobility provided by crosslinking [4].

### 3.2. Thermal Behavior

**Figure 12:**
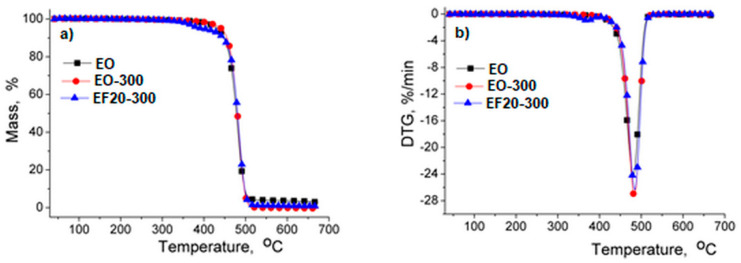
(**a**) TG and (**b**) DTG curves for EPDM samples under nitrogen atmosphere.

Figure 12 presents the TG and the corresponding derivative (DTG) curves of E0 and of 300 kGy irradiated composites E0 and EF20 in nitrogen atmosphere at a heating rate of 10 °C/min. The TGA of the EPDM/flax fiber formulations showed practically a similar behavior, the incorporation of the flax fibers in the EPDM matrix did not change significantly the thermal degradation parameters of the composites due to the uniform dispersion of the flax fibers in polymer composite which permits adding a higher fiber level. The thermal stability of the irradiated composites was slightly higher as compared to non-irradiated sample due to the crosslinking of the polymer chains. It can be seen that the EPDM samples exhibited one main step of thermal degradation that can be due to the decomposition of the rubber organic materials [4,14]. The maximum decomposition temperatures of EPDM samples were as follows: 468, 471 and 471 °C for E0, E0 and EF20 irradiated at 300 kGy, respectively. Also, the temperature at which the maximum amounts of gases were evolved (from Gram-Schmidt curve) corresponds to 475 °C (E0), 472 °C (E0, 300 kGy) and 480 °C (EF20, 300 kGy).

**Figure 13:**
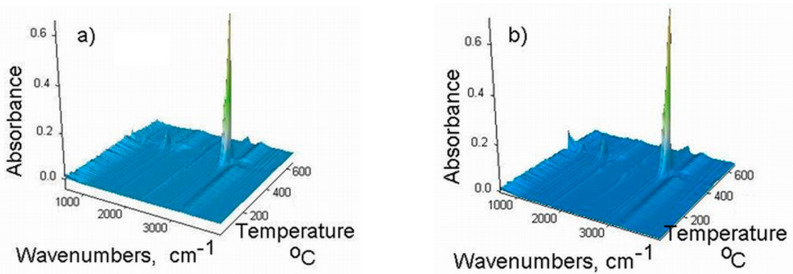
3D FTIR spectra of the evolved gases during thermal degradation for (**a**) E0; (**b**) EF20.

**Figure 14:**
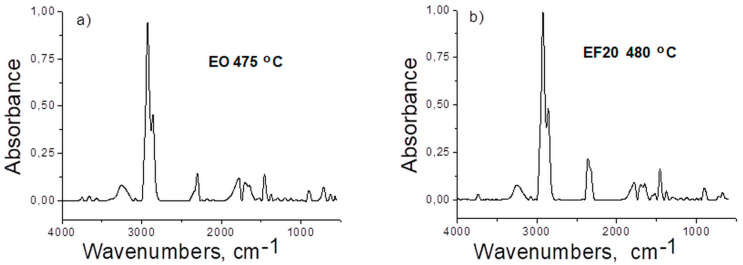
FTIR spectra of the evolved products for (**a**) E0 at 475 °C and (**b**) EF20 at 480 °C.

In order to study the thermal stability and the degradation mechanism of the EPDM and corresponding composites, TG-FTIR analysis was applied. The low molecular weight volatile compounds arising during thermal degradation were identified by TG/FTIR/MS simultaneous analysis. The 3D FTIR diagrams resulting from the thermal decomposition of the EPDM samples are presented in Figure 13. The presence of evolved gases was observed after 400 °C when the decomposition process occurs according to TG/DTG data. From the FTIR staked plot diagrams, the bidimensional FTIR spectra were extracted corresponding to the temperatures at which the degradation rate is maximum: 475 °C for E0 and 480 °C for EF20 (Figure 14). The main absorption bands are located in the proximity of the following values: 3749–2400, 3078–3010, 2925–2853, 2300–2400, 1778, 1705, 1646, 1520, 1455, 1300–1200, 1118–1064, 883–612 cm^−1^, respectively. The absorption band around 3200 cm^−1^ (ice band) is characteristic of the MCT detector (cooled with liquid nitrogen) of the TG-IR external module. The absorption bands located at 3749–3566 and 1295–1375 cm^−1^ (ν OH) can be assigned to water vapors and the bands around 2360–2300 and 668 cm^−1^ to carbon dioxide. The specific signals of the CH, CH_2_ and CH_3_ groups (νCH stretching vibrations) of aliphatic saturated derivatives correspond to the absorption bands around 2925–2853, 1375–1294, 712–720 cm^−1^ and aliphatic unsaturated derivatives (νC=C) show absorption bands in the range of 1646–1688 cm^−1^ [35,36]. Furans and furan derivatives (furan aldehyde, furanone) present stretching vibrations, νC=C, at 1570–1517 cm^−1^, νCH at 899 cm^−1^ and νC=O groups specifically of furan ring at 1124–1067 cm^−1^. The aromatic derivatives show absorption bands at 3078–3010 cm^−1^ (ν CH), ring vibrations, νC=C, at 1456 cm^−1^ and substitution bands, γCH, at 952, 821 cm^−1^.

**Figure 15:**
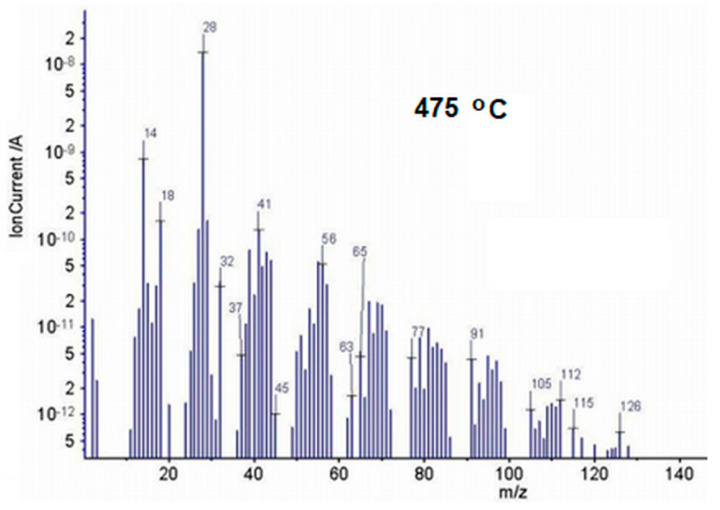
Mass spectrum of the evolved gases by thermal degradation of E0 sample, at 475 °C.

**Figure 16:**
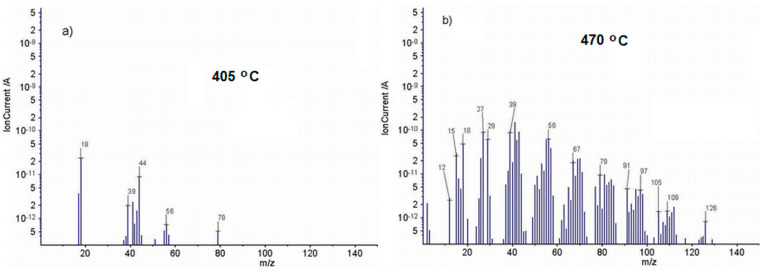
Mass spectra of the evolved gases by thermal degradation of EF20 sample at (**a**) 405 °C, (**b**) 470 °C.

MS signals represented by *m/z* values at the temperature (T_max_) corresponding to the maximum amount of released gases are given in Figure 15 and Figure 16. The chemical composition of the evolved gases during thermal degradation was determined on the basis of FTIR and mass spectra (MS) available in the NIST spectral libraries [37]. The main pyrolysis products detected by MS spectra, at a heating rate of 10 °C/min, confirmed the data obtained by the FTIR technique. The *m*/*z* signals given in Figure 15 can be associated with destruction of the EPDM polymer chains. The main ionic fragments obtained at high temperatures (475 °C) correspond to water (H_2_O^+^) *m*/*z* = 18, 17, 16; carbon dioxide (CO_2_^+^) *m*/*z* = 44, 28, 16, 12, 45; alkane and alkene derivatives such as propylene (C_3_H_6_^+^) *m*/*z* = 42, 41, 39, 27, 40, 38, 37, 26, 15); propane (C_3_H_8_^+^) *m*/*z* = 44, 29, 27,43, 39, 41, 26, 15, 42; butane (C_4_H_10_^+^) *m*/*z* = 58, 43, 29, 27, 28, 41, 39, 42, 15, 26; hexene (C_6_H_12_^+^) *m*/*z* = 84, 56, 41, 42, 55, 43, 27, 39, 69, 29; heptene (C_7_H_14_^+^) *m*/*z* = 98, 56, 41, 55, 29, 42, 70, 69, 57, 39, 27; norbornene (C_7_H_10_^+^) *m*/*z* = 94, 66, 39, 79, 67, 77, 65, 27, 40, 41; 5-ethylidene 2-norbornene (C_9_H_12_^+^) *m*/*z* = 120, 66, 91,105, 78, 39, 77, 79, 65, 92; trimethylcyclohexane (C_9_H_18_^+^) *m*/*z* = 126, 69, 111, 55, 41, 39, 56, 70, 42, 112; aromatic derivatives such as, benzene (C_6_H_6_^+^) *m*/*z* 78, 77, 51, 50, 52, 39, 70, 76, 38 and toluene (C_7_H_8_^+^) *m*/*z* = 92, 91, 65, 39, 63, 51, 93, 50, 89, 62.

In the MS spectra of EPDM/flax composite (EF20) an additional process of thermal degradation occurring up to 400 °C was found. In this range water, carbon dioxide as well as ionic fragments of some aliphatic (C_4_H_8_^+^) or aromatic (C_6_H_7_^+^) derivatives can appear (Figure 16a). Besides, the majority of MS signals evidenced for sample E0 was also identified for sample EF20 excepting the signals with *m*/*z* = 120 (Figure 16b). On the other hand, during thermal decomposition of EPDM/flax composite at 475 °C different ionic fragments provided by flax fibers containing waxes, greases, lignin, hemicellulose and pectic compounds can appear. These products can generate by pyrolysis processes different ionic fragments, such as furan (C_4_H_4_O^+^) *m*/*z* = 68, 39, 38, 40, 29, 37, 42, 60; furan aldehyde (C_5_H_4_O_2_^+^) *m*/*z* = 96, 95, 39, 38, 29, 37, 67, 40, 97, 42; 2(5*H*)-furanone (C_4_4_4_O_2_^+^) *m*/*z* = 84, 55, 27, 26, 54, 29, 39, 38, 27; 5-methyl-2-furaldehyde (C_6_H_6_O_2_^+^) *m*/*z* = 110, 109, 53, 27, 39, 51, 81, 43, 51, 111 [35].

### 3.3. Water Sorption

**Figure 17:**
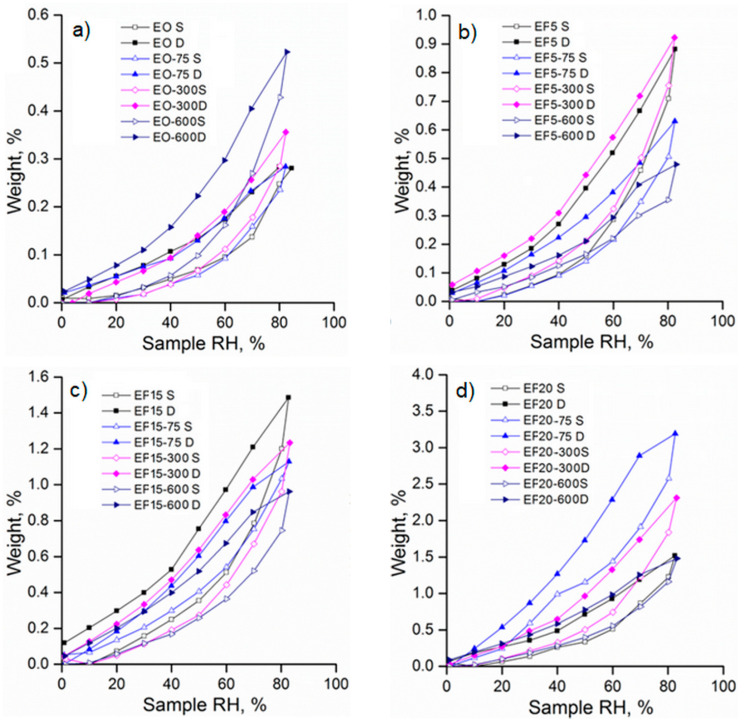
Sorption/desorption isotherms of EPDM composites (**a**) E0; (**b**) EF5; (**c**) EF15; (**d**) EF20 (S—sorption, D—desorption).

The sorption/desorption isotherms are illustrated in Figure 17. According to the IUPAC classification, these isotherms can be considered as type IV with H2 hysteresis loops characteristic of pores with an irregular array of shapes and sizes, illustrating the adsorption behavior of mesoporous materials [38,39].

Different methods have been developed to estimate the diffusion coefficients by solving the second Fick’s equations [40,41]:

(4) $$\frac{M_{t}}{M_{\infty }}=\frac{4}{h}{\left(\frac{Dt}{\pi }\right)}^{0.5}+\frac{8}{h}{\left(Dt\right)}^{0.5}\overset{\infty }{\underset{n=1}{\sum }}{\left(-1\right)}^{n}ierfc\frac{nh}{2{\left(Dt\right)}^{0.5}}$$

(5) $$\frac{M_{t}}{M_{\infty }}=1-\frac{8}{\pi^{2}}\overset{\infty }{\underset{n=0}{\sum }}\frac{1}{{\left(2n+1\right)}^{2}}e^{\frac{-{\left(2n+1\right)}^{2}\pi^{2}Dt}{h}}$$

where *t* is the time measured when the concentration was changed, *h* represents thickness of the materials, *M_o_* is the initial equilibrium mass, *M_t_ = M_o_ – M_to…t__∞_* denotes the change in mass from *M_o_* to the new equilibrium mass *M_t_*, *ierfc* represents the integral of the error function complement.

Taking into account the approximation of the first terms in Equations (4) and (5), at sufficiently short times, *M_t_*/*M_∞_* < 0.5 and sufficiently long times, *M_t_*/*M_∞_* > 0.5, one can write that:

(6) $${\left(\frac{M_{t}}{M_{\infty }}\right)}^{2}=\frac{16D_{1}}{\pi h^{2}}t=K_{1}t$$

where:

(7) $$K_{1}=-\frac{16D_{1}}{\pi h^{2}}\Rightarrow D_{1}=-\frac{K_{1}\pi h^{2}}{16}$$

(8) $$\ln\left(1-\frac{M_{t}}{M_{\infty }}\right)=\ln\frac{8}{\pi^{2}}-\frac{D_{2}\pi^{2}}{h^{2}}t=K_{2}t$$

where:

(9) $$K_{2}=-\frac{D_{2}\pi^{2}}{h^{2}}\Rightarrow D_{2}=-\frac{K_{2}h^{2}}{\pi^{2}}$$

Using the above diffusion models the results regarding the water uptake for EPDM/flax fiber composites are presented in Table 2. The Brunauer-Emmet-Teller (BET) model was chosen to fit the water sorption isotherms [42]:

(10) $$W=\frac{W_{m}\cdot C\cdot p/p_{0}}{\left(1-p/p_{0}\right)\cdot \left(1-p/p_{0}+C\cdot p/p_{0}\right)}$$

where *W* is the weight of adsorbed water, *W_m_* is the weight of water forming a monolayer, *C* represents the sorption constant and *p*/*p*_o_ is the relative humidity. The BET model allows one to calculate the surface area in a range of activities from 0.00 to 0.35. The BET data are summarized in Table 2.

Assuming cylindrical pore geometry, the average pore size was estimated using the relation (6) [43] (Table 2):

(11) $$r_{pm}=\frac{2n}{100\cdot \rho_{a}\cdot A}$$

where *r_pm_* is the average pore size, *A* denotes the BET surface area, *n* is the percentage uptake, *ρ_a_* is the solvent phase density.

As was expected, the water absorption in nonirradiated EPDM composites increases as the level of flax fiber increased (Table 2) attaining the highest water uptake of 1.51% for sample EF20. The increased water uptake could be due to the hydrophilic nature of the flax fibers. As a result, the increase of the OH groups in samples determines more hydrogen bonds between water molecules and flax fibers leading to a net weight gain of the EPDM/flax composites [44]. Also, for the EPDM composite without flax fiber the sorption capacity increases as the irradiation dose increases (Figure 17a). It was observed for electron beam-treated EPDM/flax composites EF5 and EF 15 that the sorption capacity was lower as compared to the nonirradiated sample (Table 2). The effect of electron beam irradiation was very significant for sample EF5 at 600 kGy, when the sorption capacity was found to be 0.48%. The decrease in water uptake with increasing irradiation dose can be determined by the increase of the crosslinking density during irradiation leading to the reduction of the volume required for water molecule diffusion. However, for EPDM/flax composite EF20 the water uptake and the specific surface area are accompanied with an increase of their values regardless of the irradiation dose (Table 2). Generally, the values of diffusion coefficients remain at the same order of magnitude, indicating that irradiation dose does not drastically affect the inner morphology. As expected D2 is one order of magnitude higher that D1 due to long time exposure to water vapor sorption.

## 4. Conclusions

The dielectric parameters such as dielectric constant and dielectric loss monotonically decreased with the increase of frequency, but they become independent as the frequency increased above 10^3^ Hz. The dielectric constant and dielectric loss increased with increasing flax fiber loading due to the increase of the number of polar groups in the composites leading to a higher orientation polarization. Overall, the EPDM/flax composites revealed higher values of dielectric parameters as compared to a pristine sample. The dielectric relaxation behavior of EPDM/flax composites exhibits several relaxation processes as the temperature increases, namely β-relaxation which corresponds to local motions occurring at lower temperatures and α, α′-relaxations associated to the cooperative reorientation motions of the polymer segments. The activation energy of the α′-relaxation was estimated using Vogel-Fulcher-Tammann relation having higher values for electron beam-irradiated samples. The water uptake of EPDM composites was influenced by the flax level and the irradiation dose. The simultaneous analysis of the thermal degradation of the EPDM/flax composites by TG/FTIR/MS technique showed details about the polymer decomposition process. The main gaseous products evolved during the composite degradation were determined. By tuning composition and crosslink density the desired properties of EPDM/flax fiber composites can be achieved in order to be used as polymeric insulators.

## Figures and Tables

**Table 1:** Formulation of EPDM composites.

Material/Code	Loading (phr *)				
	E0	EF5	EF10	EF15	EF20
EPDM	100	100	100	100	100
Flax	0	5	10	15	20
PEG 400	3	3	3	3	3
Irganox 1010	1	1	1	1	1
Perkadox 14-40B	8	8	8	8	8

* parts per hundred rubber.

**Table 2:** Main data evaluated from sorption isotherms and diffusion coefficients.

Sample	Sorption Capacity, % d.b.	Average Pore Size, nm	BET Data		D_1_, cm^2^ s^−1^ (M_t_/M_∞_)^2^ < 0.5	D_2_, cm^2^ s^−1^ (M_t_/M_∞_)^2^ > 0.5
			Area, m^2^ g^−1^	Monolayer, g g^−1^		
E0	0.28	3.07	1.827	0.00052	1.68 × 10^−8^	5.32 × 10^−8^
E0-75	0.29	3.83	1.519	0.00320	5.30 × 10^−8^	1.24 × 10^−7^
E0-300	0.35	5.12	1.370	0.00390	5.67 × 10^−8^	1.64 × 10^−7^
EP0-600	0.52	1.19	8.782	0.00250	5.28 × 10^−8^	1.26 × 10^−7^
EF5	0.88	2.10	8.416	0.00234	4.92 × 10^−8^	1.27 × 10^−7^
EF5-75	0.63	2.14	5.900	0.00168	4.59 × 10^−8^	1.40 × 10^−7^
EF5-300	0.92	1.72	10.700	0.00305	4.59 × 10^−8^	1.35 × 10^−7^
EF5-600	0.48	1.11	8.680	0.00240	6.80 × 10^−8^	1.32 × 10^−7^
EF15	1.48	1.56	19.050	0.00540	4.60 × 10^−8^	1.30 × 10^−7^
EF15-75	1.12	1.36	16.554	0.00471	4.29 × 10^−8^	1.56 × 10^−7^
EF15-300	0.82	2.05	8.039	0.00229	4.32 × 10^−8^	1.42 × 10^−7^
EF15-600	0.96	2.08	9.280	0.00264	4.69 × 10^−8^	1.59 × 10^−7^
EF20	1.51	0.72	42.090	0.00110	4.84 × 10^−8^	1.05 × 10^−7^
EF20-75	3.19	2.54	25.180	0.00730	4.54 × 10^−8^	1.72 × 10^−7^
EF20-300	2.31	1.47	31.617	0.00900	4.96 × 10^−8^	1.43 × 10^−7^
EF20-600	1.47	1.60	18.430	0.00520	4.91 × 10^−8^	1.47 × 10^−7^

## Data Availability

The data that support the findings of this study are available from the corresponding author upon reasonable request.
